# Supplementary material for: What do families want to improve in the management of paediatric febrile neutropenia during anti-cancer treatment? Report of a patient/public involvement group
Source: BMJ Paediatr Open. 2019 Mar 1;3(1):e000398. doi: 10.1136/bmjpo-2018-000398 (PMC6422240; doi:10.1136/bmjpo-2018-000398)
Supplement: Author's manuscript [file bmjpo-2018-000398.draft_revisions.pdf]

BMJ Paediatrics Open

BMJ Paediatrics Open is committed to open peer review. As part of this commitment we make the peer review history of every article we publish publicly available.

When an article is published we post the peer reviewers' comments and the authors' responses online. We also post the versions of the paper that were used during peer review. These are the versions that the peer review comments apply to.

The versions of the paper that follow are the versions that were submitted during the peer review process. They are not the versions of record or the final published versions. They should not be cited or distributed as the published version of this manuscript.

BMJ Paediatrics Open is an open access journal and the full, final, typeset and author-corrected version of record of the manuscript is available on our site with no access controls, subscription charges or pay-per-view fees (http://bmjpaedsopen.bmj.com).

If you have any questions on BMJ Paediatrics Open's open peer review process please email info.bmjpo@bmj.com

BMJ Paediatrics Open

What do families want to improve in the management of paediatric febrile neutropenia during anti-cancer treatment? Report of a patient/public involvement group.

Journal:	*BMJ Paediatrics Open*
Manuscript ID	bmjpo-2018-000398
Article Type:	Original article
Date Submitted by the Author:	22-Oct-2018
Complete List of Authors:	Phillips, Bob; University of York, Centre for Reviews and Dissemination; Bob Phillips<br>Depani, Sarita; University of Birmingham<br>Morgan, Jess; University of York, Centre for Reviews and Dissemination
Keywords:	Data Collection, Infectious Diseases, Oncology, Patient perspective

SCHOLARONE™
Manuscripts

What do families want to improve in the management of paediatric febrile neutropenia during anti-cancer treatment? Report of a patient/public involvement group.

Authors

*Bob Phillips [1,2], Sarita Despani [3], Jess Morgan [1]

1. Centre for Reviews and Dissemination, University of York, York, UK

2. Department of Paediatric Oncology, Leeds Children's Hospital, Leeds, UK

3. Cancer Research UK Clinical Trials Unit (CRCTU), Edgebaston, Birmingham UK

Bob Phillips bob.phillips@york.ac.uk is the corresponding author

Abstract

Background

This study reports how parents and young people who had experience of febrile neutropenia improved the design of a trial to inform the management of this condition. Five parents, a young person who had completed treatment, and three clinician-researchers contributed.

Methods

The group formed after an invitation via social media and met via video conference. Many participants were from an existing childhood cancer parent-involvement group. The initial questions for the discussion asked about the importance of the topic, the views of the need for a trial, which important outcomes should be measured, and practical aspects which would make it easier or more difficult for people to take part in it. The conversation occurred across an entire afternoon, was audio and video recorded, transcribed, analysed, and checked by those involved. A fifth parent added to this via email.

Results

The group altered the trial structure, proposing to randomise each child to one of the two management methods through the whole of their anti-cancer treatment, rather than randomising the study sites or the child at each visit. They felt even if people declined taking part in the study in the first weeks of diagnosis, their views may change and they should be allowed to consent later. They also proposed methods of collecting patient and family important data, enriching the medical information gained in the study. Active follow-up, negotiated for each individual family, was also suggested.

Conclusion

Confidential: For Review Only

Trials improving the management of febrile neutropenia for children and young people who are undergoing anti-cancer treatments should consider individual-patient randomisation, collection of 'quality of life' and 'experience of care' aspects using digital and paper methods, engage families in shared decision making around management choices and ensure adequate supportive information is available and accessible to all patients, regardless of background, geographical location, or age.

Key Messages

What is known

- Febrile neutropenia is a common complication of childhood cancer therapy which is disruptive and resource intensive
- Trials of reduction in intensity of treatment for febrile neutropenia has previously been challenging to accept for parents
- Parent/patient and public involvement in trials has modified designs and information leaflets

What this study adds

- Parent/patient and public involvement in a proposed trial of reducing antibiotic treatment for febrile neutropenia led to changes in fundamental aspects of trial design
- Proposed outcome assessments were enhanced by experts by experience describing the burden of the treatment for febrile neutropenia and trial procedures
- Video conferencing was effective despite the participants not already being well known to each other

Introduction

The treatment of malignancies in childhood is associated, in high-income countries, with five-year survival rates in excess of 80% [1]. This is possible through the use of intensive, toxicity inducing, regimens, where one-third of deaths in this group are the result of complications of therapy rather than directly due to the disease [2 3]. The cancer treatment often produces acute complications requiring unplanned hospitalisation, disruption, distress and strain upon the young person and their family [4]. One such complication is the co-occurrence of fever in the presence of neutropenia; this combination heralds a possible overwhelming infection and is considered a medical emergency [5]. The absolute risk of death or requirement for intensive care in such episodes is low; approximately 3% [6]. The challenge for families and health care professionals is to effectively treat each episode, with minimum exposure to antibiotics and disruption of family life.

Research into episodes of febrile neutropenia, and subsequent clinical practice guidelines have emphasised the need to treat promptly, assess the risk of each episode, and treat with antibiotics chosen to address individual and local resistance patterns [5]. The methods of risk assessment and discontinuing antibiotic therapy are, however, precautionary and conservative, treating two thirds of patients with broad spectrum antibiotics unnecessarily [7]. Studies have shown that biomarkers of

Confidential: For Review Only

infection/inflammation seem to predict the risk of serious infection and its resolution, but have not been used to guide management [8]. Further refinement of the approach to febrile neutropenia has been identified as a research priority [5].

In analogous situations with critically ill or immunocompromised hosts, such as adult or neonatal intensive care units, the traditional management is similar to febrile neutropenia, with the prompt use of antibiotics and discontinuing when infection has been excluded. Procalcitonin-led guidelines have been shown to reduce exposure to antibiotics and potentially improve mortality rates [9 10].

The need to improve the management of febrile neutropenia led to the development of a research proposal to use procalcitonin, which is tested on a blood sample, to assist antibiotic decision making episodes of febrile neutropenia. Deciding how to conduct such a study, which outcomes were important to measure, how to measure them, and possible barriers and solutions to a trial, was felt to be best undertaken with the engagement of clinicians, academics, and parents and young people who had direct experience of anti-cancer treatment in childhood.

This paper reports the findings of a patient/public involvement (PPI) group of clinical academics, parents and young people convened to design a study to investigate procalcitonin-assisted decision-making in the management of febrile neutropenia in children undergoing anti-cancer therapy.

Method

A request was made on social media for parents and young people who had experience of childhood cancer therapy to consider taking part in a group to discuss the proposed trial. Volunteers were gathered, and after initially attempting a face to face meeting, a video conference platform (Zoom) was used to overcome geographical barriers. The clinical academics all met in one location; the service users took part from their own homes. One participant could not get integrated audio working, so joined the conversation via telephone and mute video. The discussion lasted 2h 15 minutes.

The session was video and audio recorded. The entire meeting was transcribed, after audio immersion, and the content thematically studied. Elements of the conversation related specifically to the design and conduct of a study were developed into themes and sub-themes. Elements related to the management of febrile neutropenia were examined in a framework derived from the themes developed in a relevant PhD [11].

The costs of the group were small; transport costs and light refreshments only for the researchers, and a small fee for the video conferencing platform. The platform and the technologies were already owned by the participants. The participants volunteered their time and did not receive payment.

As this was a patient/public engagement group of experts through experiences in the development of a study, consistent with the INVOLVE definition of public involvement in research as “research being carried out ‘with’ or ‘by’ members of the public rather than ‘to’, ‘about’ or ‘for’ them” [12] no ethical review or confirmation was required.

Results

Confidential: For Review Only

Participants

Four volunteer parents were part of the UK-based PORT (Paediatric Oncology Reference Team) organisation, which consists of parents of children and young people who had experience of childhood cancer. Each was the mother of a child who had undergone cancer treatment; two with leukaemia, two with neuroblastoma. Three of these four parent's children had died of their disease. A patient who had leukaemia when a teenager also took part in the group. Of the three clinical academics, two were higher specialist trainees in paediatric oncology, and one a Consultant, who was the only male in this group. Each member of the group had prior experience with research in children's cancer beyond participation. The discussions involved descriptions of past experiences of admissions with fever and neutropenia. The experiences were from around 2006-2017. During that time period, there has been a move to some reduction in length-of-stay and marginally more consistency between centres in the UK [13]. Additional comments were added by a fifth PORT parent via email following the video conference.

Study-specific themes

Information regarding the undertaking and conduct of the study was described under three major themes; 'importance', 'how PPI changes trialists' views' and 'practical and ethical'. The theme of 'importance' was formed by concepts of 'medical consequence', 'psychological consequence', 'impact', 'unpredictability' and 'frequency of FN'. (See Figure)

Importance: The volunteers unanimously agreed that the management of episodes of febrile neutropenia was important because of its unpredictability, frequency, and medical, psychological and social ('impact') consequences. They described particularly how variation in care across different hospitals was a source of concern to them and consistency would be a positive by-product of undertaking a trial:

> *"we've got the 20-odd centres and pretty much everybody follows the same protocols [for anti-cancer treatment]... and I think it would be really reassuring for families if the POSCUs [Paediatric Oncology Shared Care Units] you know if we knew that everybody was doing the same thing"* [P1]

'How PPI changes trialists' views' describes how PPI is important in modifying the initial, genuinely held presumptions and beliefs about the best ways to conduct such a trial for families and CYP. These beliefs were drawn from the trial development group, the group of clinicians and researchers involved in designing the trial, which has over a century of experience working in children's cancer in a variety of units and countries and specific expertise in studying supportive care in this group of patients. The PPI group altered the initial trial suggestion in the following aspects:

Allocation. The initial suggestion was for group randomisation, assigned arms to by clinical unit, rather than individual patients. However, the PPI input strongly steered towards individual randomisation, but with each individual receiving the same arm of management throughout the whole trial:

Confidential: For Review Only

> *with paediatric oncology unlike many other things... everybody is on a trial... everybody... I mean... <snip>... I think everyone just expects that their treatment may be a bit different than everyone else's...* [P2]

> *once you're randomised rather than each time coming in and by randomised each time, you're better off having "this family is procalcitonin, this family is not"* [P1]

Outcome assessment. The trial group felt direct measures of patient experience would be important, more than 'quality of life' checklists. They suggested offering a daily experience journal of some form (paper, or 'app' based electronic), and believed this would supplement and enrich the medical data collected, such as admission duration, antibiotic duration, and infective organisms.

> *capturing that idea of burden beyond the hospital based stuff and things that matter to the family,* [P3]

They described how it would be important to measure the extra resources required because of FN admissions. The word 'impact' was felt to capture this rather than 'costs'.

> *impact, because it's not just about extra costs, I mean if you're having to call in grandparents and you're having to call in favours left right and centre, <snip> I mean how many times can you ask the next door neighbours to collect your kids from school* [P1]

Active safety netting. The trialists initially felt the standard approach after discharge of responsibilities passed to the family to 'return if unwell' would be safe and acceptable, but the PPI group thought an active approach to safety-netting was necessary, but should be individually negotiated:

> *I think it needs to be more than just you phone up the hospital if you have any concerns, it should be either somebody coming around or phoning you and saying "Do you have any concerns, do you have any concerns at all" and actually if you say yes …… giving you the option of coming back or having somebody over* [P4]

Gaining consent. The trialists proposed, as with usual practice, the study would be offered once to families when the clinician believed it appropriate. The PPI group, with their experience of studies and information being exchanged, thought it would be fair to allow people to decline early on, but have the opportunity to join the study if they changed their mind as their treatment journey progressed. They also floated the idea of families approaching clinicians to join, rather than being invited.

> *I don't think I even knew what febrile neutropenia was though, at the time when we were first giving our consent to all the other things? I think that's something that possibly comes with... further on... down the line... even a week or two weeks after you've given all those other*

Confidential: For Review Only

consents. Because actually, the other consents are almost live-saving things... whereas this is a real choice... and I think that batching it in with those initial forms of consents is almost taking away your flexibility of trying to consider it whether you want to do it or not [P4]

You might also get people saying 'No' right at the beginning, if it's something they don't have to agree with, and then subsequently further on during their treatment when they can really see the how much of a headache that this can be... [giggles]

- *Do you think it's OK then to offer it twice? If somebody says no the first time?* [R1]

Yes – I think I do [P4]

Because it's not like chemo A vs chemo B, it's not … it's not crucial like... you can opt in whenever you want [P2]

The theme of 'practical and ethical considerations' included the ethical aspects of; consent, randomisation, delays introduced by undergoing the trial, equity and equality, and the sharing of trial data. The practical aspects described outcome collection, safety netting, and ensuring the veracity of information collected in the trial.

Randomisation was considered a fair and ethical approach when in clinical equipoise. Along with this, a later discovery one arm proving better than the other was not considered unethical; however if being on the study disadvantaged everyone (for example, by meaning treatment would be delayed while forms were completed) then it would not have been supported. A design which was accessible for the diversity of social, cultural and economic backgrounds of potential participants was essential. Confirmation of the scientific validity of the proposal and clinical equipoise was important to the PPI team. Prior systematic reviews with meta-analysis were felt to be a very comprehensive answer to this question.

Two of the three clinical academics have a strong interest in individual participant data meta-analysis. A question was asked about data sharing in this context, and the PPI were very enthusiastic in being involved.

Definitely share. I think the thing with paediatric oncology is that we do so many international trials together, because thankfully it is rare, but ultimately I think that... [snip] we're here ultimately to try to make things better for kids of the future and if that's part of it, and it is with these meta-analysis, then definitely. [P1]

The PPI group were concerned with ensuring the veracity of information collected during the study.

"are they [study groups] actually going to tell you the truth?" [P2]

Confidential: For Review Only

The PPI group were keen to know there would be some ways of determining if the data collected were truthful and accurate: this seems to speak of a greater public awareness being required of the nature of health research governance within the country generally.

Febrile neutropenia themes

Conversations in these discussions mapped onto the framework proposed by Morgan, developed to understand the decision-making processes involved in manging episodes of febrile neutropenia [11]. The overarching concepts she described were of the quest for certainty, attaining mutual trust, and the potential for realised discretion. These were all strongly endorsed in analysis of the group discussion.

The quest for certainty involves balancing the uncertainty of outcome of each episode of febrile neutropenia, including an appreciation of probability, the use of protocols and guidelines to manage the risk, and acknowledging the adverse elements of hospitalisation. The use of protective isolation, where the child and family are kept in a single room to avoid infections being caught from other hospitalised children, or source isolation, where the child is kept in a single room to avoid an infection they have spreading onwards to others, were viewed particularly negatively.

> *It was his cupboard – [child] called it his cupboard* [P3]

Mutual trust had been a challenge, with the group describing individual health care practitioners in whom they did not place trust, and the reciprocal of this, along with the negating of parental concerns;

> *the first time that I thought it was that they were taking too much precaution and I would have much preferred him to be at home taking tablets and things... monitored every so often.. whereas the second time I think he needed more than what he was getting... and I think we were right both times actually* [P4]

The ideal management of an episode of febrile neutropenia was one where safety was assured, hospitalisation was minimised, decisions discussed with families, and support provide at home provided as desired by the family: the potential for realised discretion. The group readily acknowledged the decisions would need to be based on a range of factors, including home-to-hospital distance and the variability between parents and families in self-expressed confidence:

> *my sort of worry is that... the responsibility is even more on the parent as well. .on top of like running the house... and its that sense of responsibility as well... like they're monitoring their child and being responsible for it... and like if something did happen would they feel guilty about it or not?* [P5]

Confidential: For Review Only

Exploring how the professionals were thinking about the episode, in terms of the likelihood of adverse outcomes and their considerations, was a strength in a shared decision making approach which had been absent in many prior experiences:

> *I think it would be really helpful,* [imitates Dr speaking] *... we think its' like this [left] or we think it's like this [right]... and then chatting... and ..... You know where you are coming from and where there is a difference and you know talking about …* [P2]

> *seem to recall being in negotiations... situations where... ringing [PTC] consultants saying "This is our situation .... can you speak to them and and so on…"* [P4]

Reflections of clinical academics

The group discussions encouraged the three clinical trialists to reflect on their previous approaches to febrile neutropenia and PPI involvement in other studies. The more experienced clinical academics had undertaken PPI before, but always on a face-to-face basis. The video conferencing allowed for a more diverse group of individuals to undertake the work, with the clinicians in the same room on one screen facilitating. The makeshift re-positioning of audio for one participant through the phone served to reduce hierarchies, with collaborative suggestions and problem solving forming an early 'win' for the group. The protocol changes suggested by the group had been unexpected, as was the emphasis on the emotional burden of physical isolation. The researchers all took away from this experience the value of listening to expert parents and young people, and considering video or telephone conferencing to allow a greater number and range of people to take part in PPI events.

Discussion

The engagement of a group of parents and an ex-patient who had experience of CYP cancer with clinician-trialists developing a study to improve the management of febrile neutropenia led to changes in the proposed design of the trial, and brought out a deeper understanding of the potential concerns of participants in such a study. The wider discussions about the nature of the experience of an episode of febrile neutropenia were congruent with prior work in the field [11]. pointing particularly to actively involve parents and young people in sharing decisions about care.

The PPI involvement altered how the trial would be structured, randomisation of each child to one of the two management methods through the whole of their anti-cancer treatment, rather than randomising the study sites or the child at each visit. The suggestion of multiple opportunities to be involved in the study was welcome, and congruent with the description of an emerging expertise and empowerment in people through the childhood cancer journey [14]. They discussed practical methods of collecting data which went beyond simple admission statistics and questionnaires, to enrich the information gained in the study. Active follow-up, with healthcare initiated contact with the family, but negotiated in light of their individual family, had not been originally considered by the trialists. The discussion also shed light on the experiences of people in being involved in treatments of episodes of

Confidential: For Review Only

febrile neutropenia, with the ideal being an individualised, negotiated approach within clear, safe, guidance, consistently used across all centres.

The expertise and prior relationships in the group members of similar situations may have enhanced the easy flow of ideas and conversations in this event. All members knew at least one other participant through in-person interactions, in similar group settings or clinical interactions. Ice-breaking activities were extremely brief, as there was little ice to be broken. Future PPI work with similar groups of people would benefit from considering holding the group conversations via a video conferencing platform. The ready availability of web-cams and front-facing cameras on phones, tablet and laptop computers, and the common use of video conversations in work and home life mean these are acceptable methods to have discussions. It may be beneficial to have a 'test run' period prior to the meeting to allow any technical challenges to be met; we would suggest a period of time when 'drop in' connections to confirm all is working well would be a sensible way forwards. A backup approach, as simple as a telephone line, is also very helpful.

The findings of this study have immediately influenced an application for a feasibility study of procalcitonin guided management of febrile neutropenia. They will also influence the ongoing development of clinical practice through dissemination through the children's and young people's professional network groups. Finally, the participants in this group have expressed a wish to be part of the steering committee of a trial addressing this issue, and one has joined the study as a co-applicant, enhancing further the study design.

Figure legend

Interaction of themes and sub-themes

Funding statement

This research received no specific grant from any funding agency in the public, commercial or not-for-profit sectors. BP was supported by an NIHR Post-doctoral fellowship: grant number PDF2014-10872.

Competing Interests

There are no competing interests to declare.

Contributorship

This study was conceived by BP and SD, and developed with the assistance of JM. The audio was transcribed and analysed by BP initially with input from JM and SD. BP drafted the paper, and was critically revised and developed by JM and SD. The PPI group read and agreed with the content of the paper. The authors very gratefully acknowledge their input into this specific work.

Confidential: For Review Only

Confidential: For Review Only

References

1. Gatta G, Botta L, Rossi S, et al. Childhood cancer survival in Europe 1999–2007: results of EUROCARE-5—a population-based study. *The Lancet Oncology* 2014;15(1):35-47. doi: https://doi.org/10.1016/S1470-2045(13)70548-5
2. Creutzig U, Zimmermann M, Reinhardt D, et al. Early deaths and treatment-related mortality in children undergoing therapy for acute myeloid leukemia: analysis of the multicenter clinical trials AML-BFM 93 and AML-BFM 98. *J Clin Oncol* 2004;22(21):4384-93. doi: 22/21/4384 [pii]10.1200/JCO.2004.01.191 [published Online First: 2004/10/30]
3. Hassan H, Rompola M, Glaser AW, et al. Validation of a classification system for treatment-related mortality in children with cancer. *BMJ Paediatrics Open* 2017;1(1):e000082. doi: 10.1136/bmjpo-2017-000082
4. Morgan JE, Cleminson J, Stewart LA, et al. Meta-ethnography of experiences of early discharge, with a focus on paediatric febrile neutropenia. *Supportive Care in Cancer* 2018;26(4):1039-50. doi: 10.1007/s00520-017-3983-2
5. Lehrnbecher T, Robinson P, Fisher B, et al. Guideline for the Management of Fever and Neutropenia in Children With Cancer and Hematopoietic Stem-Cell Transplantation Recipients: 2017 Update. *J Clin Oncol* 2017;35(18):2082-94. doi: 10.1200/JCO.2016.71.7017
6. Phillips RS, Sung L, Amman RA, et al. Predicting microbiologically defined infection in febrile neutropenic episodes in children: global individual participant data multivariable meta-analysis. *Br J Cancer* 2016;114(6):623-30. doi: 10.1038/bjc.2016.28
7. Haeusler GM, Thursky KA, Mechinaud F, et al. Predicting Infectious ComplicatioNs in Children with Cancer: an external validation study. *British Journal of Cancer* 2017;117(2):171-78. doi: 10.1038/bjc.2017.154
8. Haeusler GM, Carlesse F, Phillips RS. An Updated Systematic Review and Meta-Analysis of the Predictive Value of Serum Biomarkers in the Assessment of Fever during Neutropenia in Children with Cancer. *Pediatr Infect Dis J* 2013;32(10):e390-6. doi: 10.1097/INF.0b013e31829ae38d [published Online First: 2013/05/16]
9. de Jong E, van Oers JA, Beishuizen A, et al. Efficacy and safety of procalcitonin guidance in reducing the duration of antibiotic treatment in critically ill patients: a randomised, controlled, open-label trial. *The Lancet Infectious Diseases* 2016;16(7):819-27. doi: https://doi.org/10.1016/S1473-3099(16)00053-0
10. Stocker M, van Herk W, el Helou S, et al. Procalcitonin-guided decision making for duration of antibiotic therapy in neonates with suspected early-onset sepsis: a multicentre, randomised controlled trial (NeoPIns). *The Lancet* 2017;390(10097):871-81. doi: https://doi.org/10.1016/S0140-6736(17)31444-7
11. Morgan JE, Phillips B, Stewart LA, et al. Quest for certainty regarding early discharge in paediatric low-risk febrile neutropenia: a multicentre qualitative focus group discussion study involving patients, parents and healthcare professionals in the UK. *BMJ Open* 2018;8(5):e020324. doi: 10.1136/bmjopen-2017-020324
12. INVOLVE. What is public involvement in research? 2018 [Available from: http://www.invo.org.uk/find-out-more/what-is-public-involvement-in-research-2 accessed October 2018.
13. Morgan JE, Phillips B. Winter 2017 Children's Cancer and Leukaemia Group febrile neutropenia audit. *Arch Dis Child* 2018 doi: 10.1136/archdischild-2018-315249
14. Mu PF, Lee MY, Sheng CC, et al. The experiences of family members in the year following the diagnosis of a child or adolescent with cancer: a qualitative systematic review. *JBI Database System Rev Implement Rep* 2015;13(5):293-329. doi: 10.11124/jbisrir-2015-1698

Confidential: For Review Only

Confidential: For Review Only

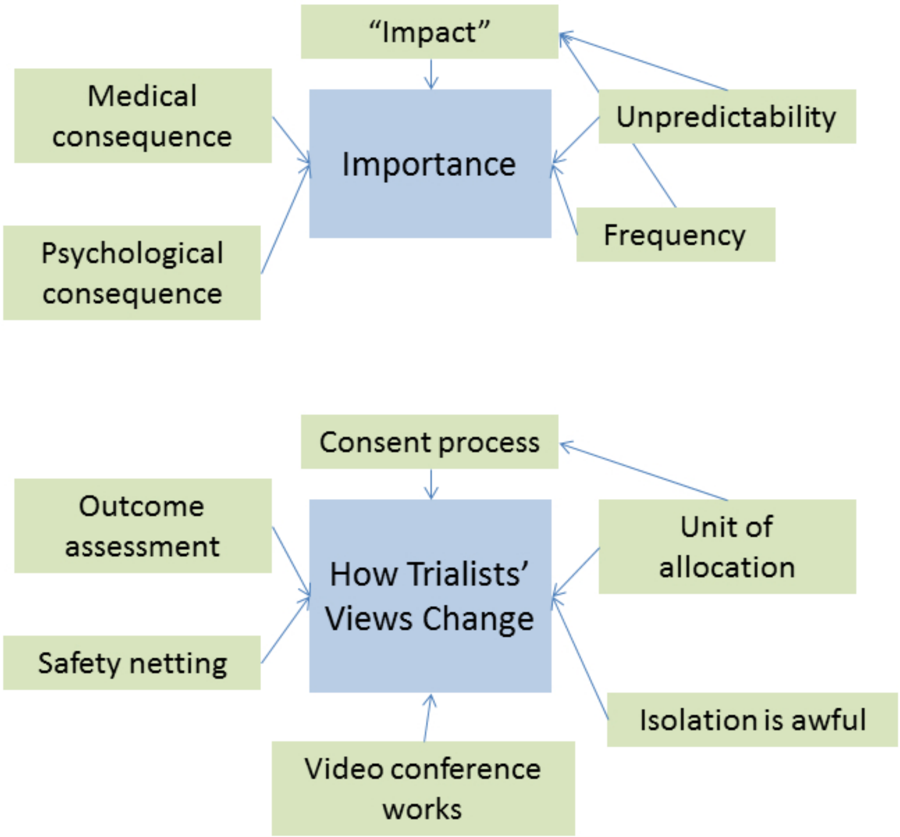

60x55mm (300 x 300 DPI)

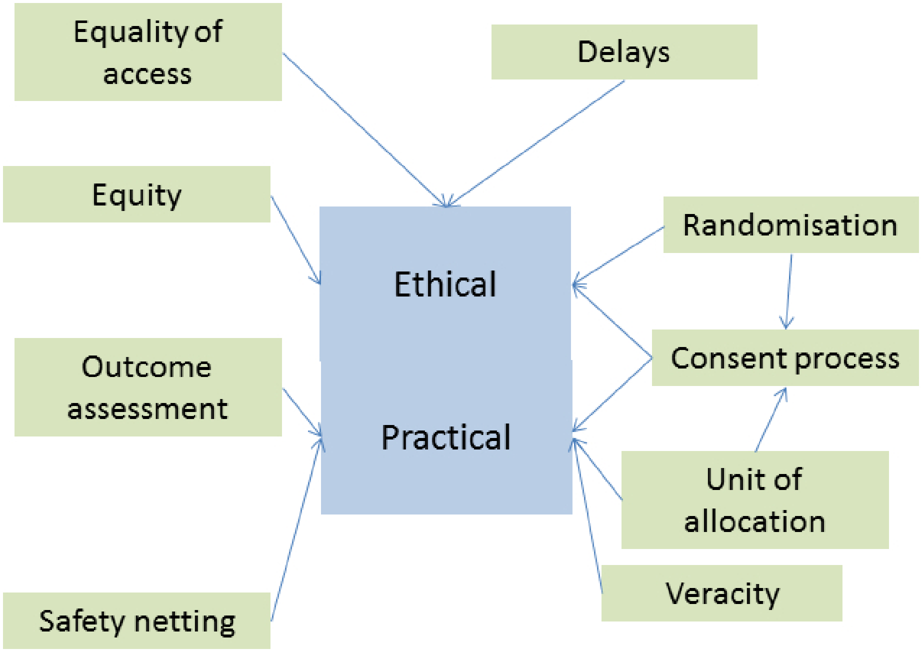

58x43mm (300 x 300 DPI)

BMJ Paediatrics Open

What do families want to improve in the management of paediatric febrile neutropenia during anti-cancer treatment? Report of a patient/public involvement group.

Journal:	*BMJ Paediatrics Open*
Manuscript ID	bmjpo-2018-000398.R1
Article Type:	Original article
Date Submitted by the Author:	06-Dec-2018
Complete List of Authors:	Phillips, Bob; University of York, Centre for Reviews and Dissemination; Leeds Childrens Hospital, Regional Department of Paediatric Haematology and Oncology<br>Depani, Sarita; University of Birmingham<br>Morgan, Jess; University of York, Centre for Reviews and Dissemination
Keywords:	Data Collection, Infectious Diseases, Oncology, Patient perspective

SCHOLARONE™
Manuscripts

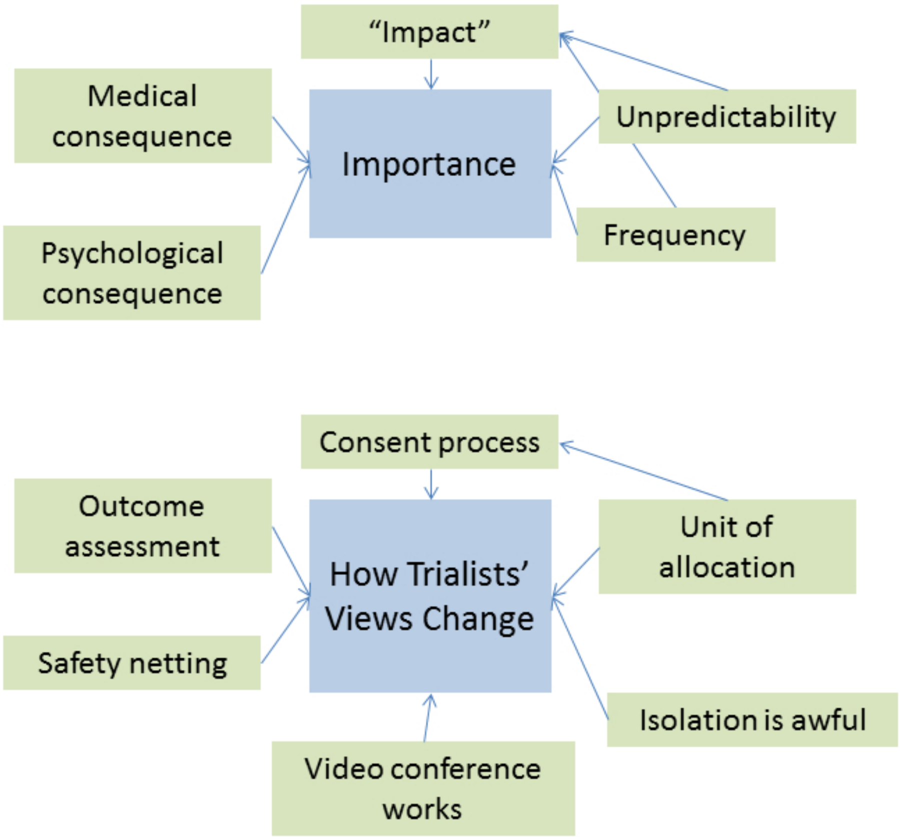

60x55mm (300 x 300 DPI)

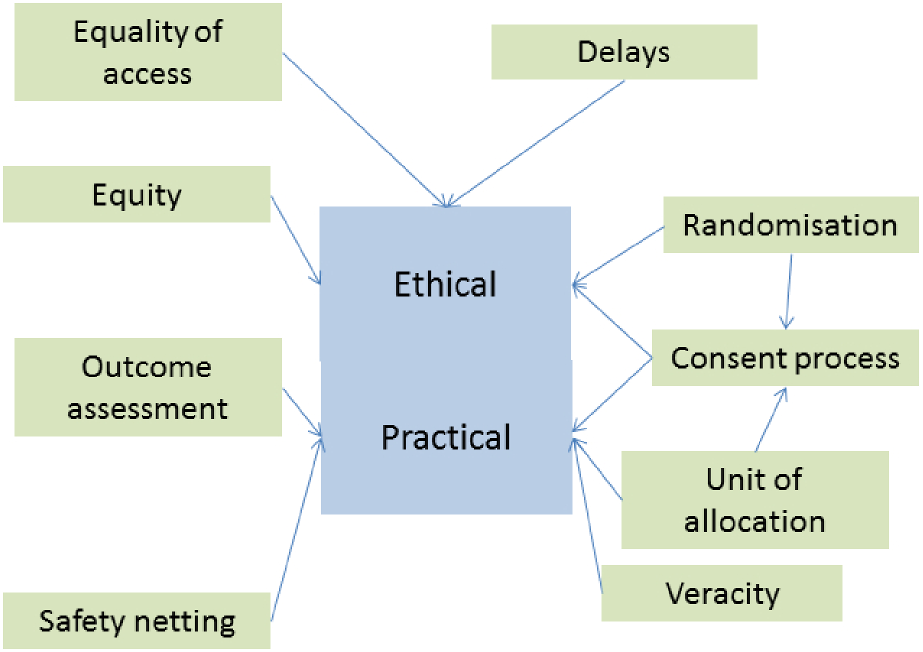

58x43mm (300 x 300 DPI)

What do families want to improve in the management of paediatric febrile neutropenia during anti-cancer treatment? Report of a patient/public involvement group.

Authors

*Bob Phillips [1,2], Sarita Depani [3], Jess Morgan [1]

1. Centre for Reviews and Dissemination, University of York, York, UK

2. Department of Paediatric Oncology, Leeds Children's Hospital, Leeds, UK

3. Cancer Research UK Clinical Trials Unit (CRCTU), Edgebaston, Birmingham UK

Bob Phillips bob.phillips@york.ac.uk is the corresponding author

Confidential: For Review Only

Abstract

Background

This study reports how parents and young people who had experience of febrile neutropenia improved the design of a trial to inform the management of this condition. Five parents, a young person who had completed treatment, and three clinician-researchers contributed.

Methods

The group formed after an invitation via social media and met via video conference. Many participants were from an existing childhood cancer parent-involvement group. The initial questions for the discussion asked about the importance of the topic, the views of the need for a trial, which important outcomes should be measured, and practical aspects which would make it easier or more difficult for people to take part in it. The conversation occurred across an entire afternoon, was audio and video recorded, transcribed, analysed, and checked by those involved. The fifth parent added to this via email.

Results

The group altered the trial structure, proposing to randomise each child to one of the two management methods through the whole of their anti-cancer treatment, rather than randomising the study sites or the child at each visit. They felt even if people declined taking part in the study in the first weeks of diagnosis, their views may change and they should be allowed to consent later. They also proposed methods of collecting patient and family important data, enriching the medical information gained in the study. Active follow-up, negotiated for each individual family, was also suggested.

Conclusion

Trials improving the management of febrile neutropenia for children and young people who are undergoing anti-cancer treatments should consider individual-patient randomisation, collection of

‘quality of life’ and ‘experience of care’ aspects using digital and paper methods, engage families in shared decision making around management choices and ensure adequate supportive information is available and accessible to all patients, regardless of background, geographical location, or age.

Key Messages

What is known

- Febrile neutropenia is a common complication of childhood cancer therapy which is disruptive and resource intensive
- Trials of reduction in intensity of treatment for febrile neutropenia has previously been challenging to accept for parents
- Parent/patient and public involvement in trials has improved study designs, research comprehension and engagement materials

What this study adds

- Parent/patient and public involvement in a proposed trial of reducing antibiotic treatment for febrile neutropenia led to changes in fundamental aspects of trial design
- Proposed outcome assessments were enhanced by experts by experience describing the burden of the treatment for febrile neutropenia and trial procedures
- Video conferencing for parent/patient and public involvement was effective despite the participants not already being well known to each other

Introduction

The treatment of malignancies in childhood is associated, in high-income countries, with five-year survival rates in excess of 80% [1]. This is possible through the use of intensive, toxicity inducing, regimens, where one-third of deaths in this group are the result of complications of therapy rather than directly due to the disease [2 3]. The cancer treatment often produces acute complications requiring unplanned hospitalisation, disruption, distress and strain upon the young person and their family [4]. One such complication is the co-occurrence of fever in the presence of neutropenia; this combination heralds a possible overwhelming infection and is considered a medical emergency [5]. The absolute risk of death or requirement for intensive care in such episodes is low; approximately 3% [6]. The challenge for families and health care professionals is to effectively treat each episode, with minimum exposure to antibiotics and disruption of family life.

Research into episodes of febrile neutropenia, and subsequent clinical practice guidelines have emphasised the need to treat promptly, assess the risk of each episode, and treat with antibiotics chosen to address individual and local resistance patterns [5]. The methods of risk assessment and discontinuing antibiotic therapy are, however, precautionary and conservative, treating two thirds of patients with broad spectrum antibiotics unnecessarily [7]. Studies have shown that biomarkers of infection/inflammation seem to predict the risk of serious infection and its resolution, but have not been used to guide management [8]. Further refinement of the approach to febrile neutropenia has been identified as a research priority [5].

Confidential: For Review Only

In analogous situations with critically ill or immunocompromised hosts, such as adult or neonatal intensive care units, the traditional management is similar to febrile neutropenia, with the prompt use of antibiotics and discontinuing when infection has been excluded. Procalcitonin-led guidelines have been shown to reduce exposure to antibiotics and potentially improve mortality rates [9 10].

The need to improve the management of febrile neutropenia led to the development of a research proposal to use procalcitonin, which is tested on a blood sample, to assist antibiotic decision making episodes of febrile neutropenia. Deciding how to conduct such a study, which outcomes were important to measure, how to measure them, and possible barriers and solutions to a trial, was felt to be best undertaken with the engagement of clinicians, academics, and parents and young people who had direct experience of anti-cancer treatment in childhood. Previous work had shown how such involvement led to improved research focus, better interview questions, and enhanced the skills of children and young people undertaking such work[11].

This paper reports the findings of a patient/public involvement (PPI) group, where researchers, parents and young people convened to design a study to investigate procalcitonin-assisted decision-making in the management of febrile neutropenia in children undergoing anti-cancer therapy.

Method

A request was made on social media for parents and young people who had experience of childhood cancer therapy to consider taking part in a group to discuss the proposed trial. Volunteers were gathered, and after initially attempting a face to face meeting, a video conference platform (Zoom) was used to overcome geographical barriers to promote inclusiveness and working together. The researchers, all clinical doctors with additional academic roles, all met in one location; the public contributors took part from their own homes. One participant could not get integrated audio working, so joined the conversation via telephone and mute video. The discussion lasted 2h 15 minutes.

The session was structured to introduce febrile neutropenia, the existing evidence for the proposed intervention, and the rationale for a randomised feasibility study (see Box 1 for the initial plan). The PPI group all had knowledge of clinical studies, including trials, in children and young people with cancer. The discussion followed a series of questions about the experience of febrile neutropenia, it's management, the perceived challenges with current approaches and how the study would be best organised to meet these.

The session was video and audio recorded. The entire meeting was transcribed, after audio immersion, and the content thematically studied. Elements of the conversation related specifically to the design and conduct of a study were developed into themes and sub-themes. Elements related to the management of febrile neutropenia were examined in a framework derived from the themes developed in a relevant PhD [12]. Following the meeting, a summary was shared and agreed, and the full report from which this paper is derived was reviewed by the PPI group.

The costs of the group were small; transport costs and light refreshments only for the researchers, and a small fee for the video conferencing platform. The platform and the technologies were already owned by the participants. The participants volunteered their time, in line with their voluntary involvement with similar charitable activities, and did not receive payment.

Confidential: For Review Only

As this was a patient/public engagement group no ethical review was required. This is consistent with the INVOLVE definition of public involvement in research as “research being carried out ‘with’ or ‘by’ members of the public rather than ‘to’, ‘about’ or ‘for’ them” [13] , Despite the lack of a formal requirement for research ethics committee oversight, and ethical approach to such work is necessary. Such an approach has been described[14], and the key elements of a fair choice to partake in the work, appropriate training and support to understand the questions asked, making sure access was as equitable as possible and providing recognition for the work were all considered in this project.

Results

Participants

Four volunteer parents were part of the UK-based PORT (Paediatric Oncology Reference Team) organisation, which consists of parents of children and young people who had experience of childhood cancer. Each was the mother of a child who had undergone cancer treatment; two with leukaemia, two with neuroblastoma. Three of these four parent’s children had died of their disease. A patient who had leukaemia when a teenager also took part in the group, unrelated to the other participants. Of the three researchers, two were higher specialist trainees in paediatric oncology, and one a Consultant, who was the only male in this group. Each member of the group had prior experience with research in children’s cancer beyond participation. The discussions involved descriptions of past experiences of admissions with fever and neutropenia. The experiences were from around 2006-2017. During that time period, there has been a move to some reduction in length-of-stay and marginally more consistency between centres in the UK [15]. Additional comments were added by a fifth PORT parent via email following the video conference.

Study-specific themes

Information regarding the undertaking and conduct of the study was described under three major themes; ‘importance’, ‘how PPI changes researchers’ views’ and ‘practical and ethical’. The theme of ‘importance’ was formed by concepts of ‘medical consequence’, ‘psychological consequence’, ‘impact’, ‘unpredictability’ and ‘frequency of FN’. (See Figure)

Importance: The group members unanimously agreed that the management of episodes of febrile neutropenia was important because of its unpredictability, frequency, and medical, psychological and social (‘impact’) consequences. They described particularly how variation in care across different hospitals was a source of concern to them and consistency would be a positive by-product of undertaking a trial:

> *“we’ve got the 20-odd centres and pretty much everybody follows the same protocols [for anti-cancer treatment]... and I think it would be really reassuring for families if the POSCUs [Paediatric Oncology Shared Care Units] you know if we knew that everybody was doing the same thing”* [P1]

‘How PPI changes researchers’ views’ describes the impact of this work: of how PPI is important in modifying the initial, genuinely held presumptions and beliefs about the best ways to conduct such a

Confidential: For Review Only

trial for families and CYP. These beliefs were drawn from the trial development group, the group of clinicians and researchers involved in designing the trial, which has over a century of experience working in children's cancer in a variety of units and countries and specific expertise in studying supportive care in this group of patients. The impact of the group is seen in in the following aspects:

Allocation. The initial suggestion was for group randomisation, assigned arms to by clinical unit, rather than individual patients. However, the PPI input strongly steered towards individual randomisation, but with each individual receiving the same arm of management throughout the whole trial:

> *with paediatric oncology unlike many other things... everybody is on a trial... everybody... I mean... <snip>... I think everyone just expects that their treatment may be a bit different than everyone else's...* [P2]

> *once you're randomised rather than each time coming in and by randomised each time, you're better off having "this family is procalcitonin, this family is not"* [P1]

Outcome assessment. The group members felt direct measures of patient experience would be important, more than 'quality of life' checklists. They suggested offering a daily experience journal of some form (paper, or 'app' based electronic), and believed this would supplement and enrich the medical data collected, such as admission duration, antibiotic duration, and infective organisms.

> *capturing that idea of burden beyond the hospital based stuff and things that matter to the family,* [P3]

They described how it would be important to measure the extra resources required because of FN admissions. The word 'impact' was felt to capture this rather than 'costs'.

> *impact, because it's not just about extra costs, I mean if you're having to call in grandparents and you're having to call in favours left right and centre, <snip> I mean how many times can you ask the next door neighbours to collect your kids from school* [P1]

Active safety netting. The researchers initially felt the standard approach after discharge of responsibilities passed to the family to 'return if unwell' would be safe and acceptable, but the PPI group thought an active approach to safety-netting was necessary, but should be individually negotiated:

> *I think it needs to be more than just you phone up the hospital if you have any concerns, it should be either somebody coming around or phoning you and saying "Do you have any concerns, do you have any concerns at all" and actually if you say yes …… giving you the option of coming back or having somebody over* [P4]

Gaining consent. The researchers proposed, as with usual practice, the study would be offered once

Confidential: For Review Only

to families when the clinician believed it appropriate. The group members, with their experience of studies and information being exchanged, thought it would be fair to allow people to decline early on, but have the opportunity to join the study if they changed their mind as their treatment journey progressed. They also floated the idea of families approaching clinicians to join, rather than being invited.

> *I don't think I even knew what febrile neutropenia was though, at the time when we were first giving our consent to all the other things? I think that's something that possibly comes with... further on... down the line... even a week or two weeks after you've given all those other consents. Because actually, the other consents are almost live-saving things... whereas this is a real choice... and I think that batching it in with those initial forms of consents is almost taking away your flexibility of trying to consider it whether you want to do it or not* [P4]

> *You might also get people saying 'No' right at the beginning, if it's something they don't have to agree with, and then subsequently further on during their treatment when they can really see the how much of a headache that this can be... [giggles]*
>
> - *Do you think it's OK then to offer it twice? If somebody says no the first time?* [R1]
>
> *Yes – I think I do* [P4]

> *Because it's not like chemo A vs chemo B, it's not … it's not crucial like... you can opt in whenever you want* [P2]

The theme of 'practical and ethical considerations' included the ethical aspects of; consent, randomisation, delays introduced by undergoing the trial, equity and equality, and the sharing of trial data. The practical aspects described outcome collection, safety netting, and ensuring the veracity of information collected in the trial.

Randomisation was considered a fair and ethical approach when in clinical equipoise. Along with this, a later discovery one arm proving better than the other was not considered unethical; however if being on the study disadvantaged everyone (for example, by meaning treatment would be delayed while forms were completed) then it would not have been supported. A design which was accessible for the diversity of social, cultural and economic backgrounds of potential participants was essential. Confirmation of the scientific validity of the proposal and clinical equipoise was important to the PPI group. Prior systematic reviews with meta-analysis were felt to be a very comprehensive answer to this question.

Two of the three researchers have a strong interest in individual participant data meta-analysis. A question was asked about data sharing in this context, and the PPI were very enthusiastic in being involved.

Confidential: For Review Only

> *Definitely share. I think the thing with paediatric oncology is that we do so many international trials together, because thankfully it is rare, but ultimately I think that... [snip] we're here ultimately to try to make things better for kids of the future and if that's part of it, and it is with these meta-analysis, then definitely.* [P1]

The group members were concerned about study governance, for example ensuring the veracity of information collected during the study.

> *"are they [study groups] actually going to tell you the truth?"* [P2]

The members were keen to know there would be some ways of determining if the data collected were truthful and accurate: this seems to speak of a greater public awareness being required of the nature of health research governance within the country generally.

An offer for expressions of interest in continuing to engage with the study governance was enthusiastically met by the participants. One of the group has joined the funding application as a co-applicant, and has helped develop the grant application and is planned to be involved in the qualitative data collection and analysis as a co-investigator.Febrile neutropenia themes

Conversations in these discussions mapped onto the framework proposed by Morgan, developed to understand the decision-making processes involved in manging episodes of febrile neutropenia [12]. The overarching concepts she described were of the quest for certainty, attaining mutual trust, and the potential for realised discretion. These were all strongly endorsed in analysis of the group discussion.

The quest for certainty involves balancing the uncertainty of outcome of each episode of febrile neutropenia, including an appreciation of probability, the use of protocols and guidelines to manage the risk, and acknowledging the adverse elements of hospitalisation. The use of protective isolation, where the child and family are kept in a single room to avoid infections being caught from other hospitalised children, or source isolation, where the child is kept in a single room to avoid an infection they have spreading onwards to others, were viewed particularly negatively.

> *It was his cupboard – [child] called it his cupboard* [P3]

Mutual trust had been a challenge, with the group describing individual health care practitioners in whom they did not place trust, and the reciprocal of this, along with the negating of parental concerns;

> *the first time that I thought it was that they were taking too much precaution and I would have much preferred him to be at home taking tablets and things... monitored every so often.. whereas the second time I think he needed more than what he was getting... and I think we were right both times actually* [P4]

Confidential: For Review Only

The ideal management of an episode of febrile neutropenia was one where safety was assured, hospitalisation was minimised, decisions discussed with families, and support provide at home provided as desired by the family: the potential for realised discretion. The group readily acknowledged the decisions would need to be based on a range of factors, including home-to-hospital distance and the variability between parents and families in self-expressed confidence:

> *my sort of worry is that... the responsibility is even more on the parent as well. .on top of like running the house... and its that sense of responsibility as well... like they're monitoring their child and being responsible for it... and like if something did happen would they feel guilty about it or not?* [P5]

Exploring how the professionals were thinking about the episode, in terms of the likelihood of adverse outcomes and their considerations, was a strength in a shared decision making approach which had been absent in many prior experiences:

> *I think it would be really helpful,* [imitates Dr speaking] *... we think its' like this [left] or we think it's like this [right]... and then chatting... and ..... You know where you are coming from and where there is a difference and you know talking about …* [P2]

> *seem to recall being in negotiations... situations where... ringing [PTC] consultants saying "This is our situation .... can you speak to them and and so on…"* [P4]

Reflections of clinical academics

The group discussions encouraged the three researchers to reflect on their previous approaches to febrile neutropenia and PPI involvement in other studies. The more experienced researchers had undertaken PPI before, but always on a face-to-face basis. The video conferencing allowed for a more diverse group of individuals to undertake the work, with the researchers in the same room on one screen facilitating. The makeshift re-positioning of audio for one participant through the phone served to reduce hierarchies, with collaborative suggestions and problem solving forming an early 'win' for the group. This method worked well with the age and technological skills of this group, but may be less successful if a group with fewer technology skills or younger age were being involved. The protocol changes suggested by the group had been unexpected, as was the emphasis on the emotional burden of physical isolation. The researchers all took away from this experience the value of listening to expert parents and young adults, and considering video or telephone conferencing to allow a greater number and range of people to take part in PPI events.

Discussion

The engagement of a group of parents and an ex-patient who had experience of CYP cancer with researchers developing a study to improve the management of febrile neutropenia led to changes in the proposed design of the trial, and brought out a deeper understanding of the potential concerns of participants in such a study. The wider discussions about the nature of the experience of an episode of febrile neutropenia were congruent with prior work in the field [12] pointing particularly to actively involve parents and young people in sharing decisions about care.

Confidential: For Review Only

The PPI involvement altered how the trial would be structured, randomisation of each child to one of the two management methods through the whole of their anti-cancer treatment, rather than randomising the study sites or the child at each visit (see Box 2 for specific changes). The suggestion of multiple opportunities to be involved in the study was welcome, and congruent with the description of an emerging expertise and empowerment in people through the childhood cancer journey [16]. They discussed practical methods of collecting data which went beyond simple admission statistics and questionnaires, to enrich the information gained in the study. Active follow-up, with healthcare initiated contact with the family, but negotiated in light of their individual family, had not been originally considered by the researchers. The discussion also shed light on the experiences of people in being involved in treatments of episodes of febrile neutropenia, with the ideal being an individualised, negotiated approach within clear, safe, guidance, consistently used across all centres.

The expertise and prior relationships in the group members of similar situations may have enhanced the easy flow of ideas and conversations in this event. All members knew at least one other participant through in-person interactions, in similar group settings or clinical interactions. Ice-breaking activities were extremely brief, as there was little ice to be broken. None of the participants were paid for their time undertaking this work. The INVOLVE guidelines suggest involvement should come with re-imbursement, the group all undertook work with charities related to childhood cancer treatment, research and support and saw this as an extension of their other activities. Future PPI work with similar groups of people would benefit from considering holding the group conversations via a video conferencing platform. The ready availability of web-cams and front-facing cameras on phones, tablet and laptop computers, and the common use of video conversations in work and home life mean these were acceptable methods to have discussions with this group, though may not work with younger children or those unfamiliar with video conferencing. If the approach is used, it may be beneficial to have a ‘test run’ period prior to the meeting to allow any technical challenges to be met; we would suggest a period of time when ‘drop in’ connections to confirm all is working well would be a sensible way forwards. A backup approach, as simple as a telephone line, is also very helpful. Direct advertising of the chance to be involved in the work to young people via other groups, such as Young People’s Advisory Groups hosted by organisations such as the National Cancer Research Institute or Clic-Sergant, or advertising through the Teenage Cancer Trust, may have meant more than one young person was involved.

The findings of this study have immediately influenced an application for a feasibility study of procalcitonin guided management of febrile neutropenia. They will also influence the ongoing development of clinical practice through dissemination through the children’s and young people’s professional network groups. The participants in this group have expressed a wish to be part of the steering committee of a trial addressing this issue, and the ongoing study development will also seek further young people to be involved, following INVOLVE guidelines[17 18]. One of the group members has joined the study as a co-applicant, developing the grant, and plans to be involved as a co-investigator..

Figure legend

Interaction of themes and sub-themes

Confidential: For Review Only

Funding statement

This research received no specific grant from any funding agency in the public, commercial or not-for-profit sectors. BP was supported by an NIHR Post-doctoral fellowship: grant number PDF2014-10872.

Competing Interests

There are no competing interests to declare.

Contributorship

This study was conceived by BP and SD, and developed with the assistance of JM. The audio was transcribed and analysed by BP initially with input from JM and SD. BP drafted the paper, and was critically revised and developed by JM and SD. The PPI group read and agreed with the content of the paper. The authors very gratefully acknowledge their input into this specific work.

Box 1

Site-randomised trial (randomising by hospital) using cluster or step-wedge approach

Sites consented

Use of single quality of life questionnaire at discharge

Patient contact for trial purposes only to occur while in-patient – not after discharge

Antibiotic decision making on procalcitonin measurements and clinical judgement without family involvement

Box 2

Individual patient randomised trial (randomising by patient, not by episode)

Consent permissible at any point during the cancer journey while still 'at risk'

Richer patient experience measures – not just patient QoL but family experience and their costs to be captured

Active follow-up after discharge

Explicitly encouraging shared decision making and sharing of results with families to decide antibiotic use

Confidential: For Review Only

Confidential: For Review Only

References

1. Gatta G, Botta L, Rossi S, et al. Childhood cancer survival in Europe 1999–2007: results of EUROCARE-5—a population-based study. *The Lancet Oncology* 2014;15(1):35-47. doi: https://doi.org/10.1016/S1470-2045(13)70548-5
2. Creutzig U, Zimmermann M, Reinhardt D, et al. Early deaths and treatment-related mortality in children undergoing therapy for acute myeloid leukemia: analysis of the multicenter clinical trials AML-BFM 93 and AML-BFM 98. *J Clin Oncol* 2004;22(21):4384-93. doi: 22/21/4384 [pii]

10.1200/JCO.2004.01.191 [published Online First: 2004/10/30]
3. Hassan H, Rompola M, Glaser AW, et al. Validation of a classification system for treatment-related mortality in children with cancer. *BMJ Paediatrics Open* 2017;1(1):e000082. doi: 10.1136/bmjpo-2017-000082
4. Morgan JE, Cleminson J, Stewart LA, et al. Meta-ethnography of experiences of early discharge, with a focus on paediatric febrile neutropenia. *Supportive Care in Cancer* 2018;26(4):1039-50. doi: 10.1007/s00520-017-3983-2
5. Lehrnbecher T, Robinson P, Fisher B, et al. Guideline for the Management of Fever and Neutropenia in Children With Cancer and Hematopoietic Stem-Cell Transplantation Recipients: 2017 Update. *J Clin Oncol* 2017;35(18):2082-94. doi: 10.1200/JCO.2016.71.7017
6. Phillips RS, Sung L, Amman RA, et al. Predicting microbiologically defined infection in febrile neutropenic episodes in children: global individual participant data multivariable meta-analysis. *Br J Cancer* 2016;114(6):623-30. doi: 10.1038/bjc.2016.28
7. Haeusler GM, Thursky KA, Mechinaud F, et al. Predicting Infectious ComplicatioNs in Children with Cancer: an external validation study. *British Journal of Cancer* 2017;117(2):171-78. doi: 10.1038/bjc.2017.154
8. Haeusler GM, Carlesse F, Phillips RS. An Updated Systematic Review and Meta-Analysis of the Predictive Value of Serum Biomarkers in the Assessment of Fever during Neutropenia in Children with Cancer. *Pediatr Infect Dis J* 2013;32(10):e390-6. doi: 10.1097/INF.0b013e31829ae38d [published Online First: 2013/05/16]
9. de Jong E, van Oers JA, Beishuizen A, et al. Efficacy and safety of procalcitonin guidance in reducing the duration of antibiotic treatment in critically ill patients: a randomised, controlled, open-label trial. *The Lancet Infectious Diseases* 2016;16(7):819-27. doi: https://doi.org/10.1016/S1473-3099(16)00053-0
10. Stocker M, van Herk W, el Helou S, et al. Procalcitonin-guided decision making for duration of antibiotic therapy in neonates with suspected early-onset sepsis: a multicentre, randomised controlled trial (NeoPIns). *The Lancet* 2017;390(10097):871-81. doi: https://doi.org/10.1016/S0140-6736(17)31444-7
11. Bird D, Culley L, Lakhanpaul M. Why collaborate with children in health research: an analysis of the risks and benefits of collaboration with children. *Archives of disease in childhood - Education & practice edition* 2013;98(2):42-48. doi: 10.1136/archdischild-2012-303470
12. Morgan JE, Phillips B, Stewart LA, et al. Quest for certainty regarding early discharge in paediatric low-risk febrile neutropenia: a multicentre qualitative focus group discussion study involving patients, parents and healthcare professionals in the UK. *BMJ Open* 2018;8(5):e020324. doi: 10.1136/bmjopen-2017-020324
13. INVOLVE. What is public involvement in research? 2018 [Available from: http://www.invo.org.uk/find-out-more/what-is-public-involvement-in-research-2 accessed October 2018.
14. Mitchell SJ, Slowther A-M, Coad J, et al. Ethics and patient and public involvement with children and young people. *Archives of disease in childhood - Education & practice edition* 2018 doi: 10.1136/archdischild-2017-313480
15. Morgan JE, Phillips B. Winter 2017 Children's Cancer and Leukaemia Group febrile neutropenia audit. *Arch Dis Child* 2018 doi: 10.1136/archdischild-2018-315249

Confidential: For Review Only

16. Mu PF, Lee MY, Sheng CC, et al. The experiences of family members in the year following the diagnosis of a child or adolescent with cancer: a qualitative systematic review. *JBI Database System Rev Implement Rep* 2015;13(5):293-329. doi: 10.11124/jbisrir-2015-1698
17. INVOLVE. Guidance on the use of social media to actively involve people in research: INVOLVE, 2017.
18. INVOLVE. Involving children and young people in research: top tips and essential key issues for researchers 2016: INVOLVE, 2016.

Confidential: For Review Only

BMJ Paediatrics Open

What do families want to improve in the management of paediatric febrile neutropenia during anti-cancer treatment? Report of a patient/public involvement group.

Journal:	*BMJ Paediatrics Open*
Manuscript ID	bmjpo-2018-000398.R2
Article Type:	Original article
Date Submitted by the Author:	09-Jan-2019

Complete List of Authors:	Phillips, Bob; University of York, Centre for Reviews and Dissemination; Leeds Childrens Hospital, Regional Department of Paediatric Haematology and Oncology<br>Depani, Sarita; University of Birmingham<br>Morgan, Jess; University of York, Centre for Reviews and Dissemination

Keywords:	Data Collection, Infectious Diseases, Oncology, Patient perspective

SCHOLARONE™
Manuscripts

What do families want to improve in the management of paediatric febrile neutropenia during anti-cancer treatment? Report of a patient/public involvement group.

Authors

*Bob Phillips [1,2], Sarita Depani [3], Jess Morgan [1]

1. Centre for Reviews and Dissemination, University of York, York, UK

2. Department of Paediatric Oncology, Leeds Children's Hospital, Leeds, UK

3. Cancer Research UK Clinical Trials Unit (CRCTU), Edgebaston, Birmingham UK

Bob Phillips bob.phillips@york.ac.uk is the corresponding author

Confidential: For Review Only

Abstract

Background

This study reports how parents and young people who had experience of febrile neutropenia improved the design of a trial to inform the management of this condition. Five parents, a young person who had completed treatment, and three clinician-researchers contributed.

Methods

The group formed after an invitation via social media and met via video conference. Many participants were from an existing childhood cancer parent-involvement group. The initial questions for the discussion asked about the importance of the topic, the views of the need for a trial, which important outcomes should be measured, and practical aspects which would make it easier or more difficult for people to take part in it. The conversation occurred across an entire afternoon, was audio and video recorded, transcribed, analysed, and checked by those involved. The fifth parent added to this via email.

Results

The group altered the trial structure, proposing to randomise each child to one of the two management methods through the whole of their anti-cancer treatment, rather than randomising the study sites or the child at each visit. They felt even if people declined taking part in the study in the first weeks of diagnosis, their views may change and they should be allowed to consent later. They also proposed methods of collecting patient and family important data, enriching the medical information gained in the study. Active follow-up, negotiated for each individual family, was also suggested.

Conclusion

Trials improving the management of febrile neutropenia for children and young people who are undergoing anti-cancer treatments should consider individual-patient randomisation, collection of

'quality of life' and 'experience of care' aspects using digital and paper methods, engage families in shared decision making around management choices and ensure adequate supportive information is available and accessible to all patients, regardless of background, geographical location, or age.

Key Messages

What is known

- Febrile neutropenia is a common complication of childhood cancer therapy which is disruptive and resource intensive
- Trials of reduction in intensity of treatment for febrile neutropenia has previously been challenging to accept for parents
- Parent/patient and public involvement in trials has improved study designs, research comprehension and engagement materials

What this study adds

- Parent/patient and public involvement in a proposed trial of reducing antibiotic treatment for febrile neutropenia led to changes in fundamental aspects of trial design
- Proposed outcome assessments were enhanced by experts by experience describing the burden of the treatment for febrile neutropenia and trial procedures
- Video conferencing for parent/patient and public involvement was effective despite the participants not already being well known to each other

Introduction

The treatment of malignancies in childhood is associated, in high-income countries, with five-year survival rates in excess of 80% [1]. This is possible through the use of intensive, toxicity inducing, regimens, where one-third of deaths in this group are the result of complications of therapy rather than directly due to the disease [2 3]. The cancer treatment often produces acute complications requiring unplanned hospitalisation, disruption, distress and strain upon the young person and their family [4]. One such complication is the co-occurrence of fever in the presence of neutropenia; this combination heralds a possible overwhelming infection and is considered a medical emergency [5]. The absolute risk of death or requirement for intensive care in such episodes is low; approximately 3% [6]. The challenge for families and health care professionals is to effectively treat each episode, with minimum exposure to antibiotics and disruption of family life.

Research into episodes of febrile neutropenia, and subsequent clinical practice guidelines have emphasised the need to treat promptly, assess the risk of each episode, and treat with antibiotics chosen to address individual and local resistance patterns [5]. The methods of risk assessment and discontinuing antibiotic therapy are, however, precautionary and conservative, treating two thirds of patients with broad spectrum antibiotics unnecessarily [7]. Studies have shown that biomarkers of infection/inflammation seem to predict the risk of serious infection and its resolution, but have not been used to guide management [8]. Further refinement of the approach to febrile neutropenia has been identified as a research priority [5].

Confidential: For Review Only

In analogous situations with critically ill or immunocompromised hosts, such as adult or neonatal intensive care units, the traditional management is similar to febrile neutropenia, with the prompt use of antibiotics and discontinuing when infection has been excluded. Procalcitonin-led guidelines have been shown to reduce exposure to antibiotics and potentially improve mortality rates [9 10].

The need to improve the management of febrile neutropenia led to the development of a research proposal to use procalcitonin, which is tested on a blood sample, to assist antibiotic decision making episodes of febrile neutropenia. Deciding how to conduct such a study, which outcomes were important to measure, how to measure them, and possible barriers and solutions to a trial, was felt to be best undertaken with the engagement of clinicians, academics, and parents and young people who had direct experience of anti-cancer treatment in childhood. Previous work had shown how such involvement led to improved research focus, better interview questions, and enhanced the skills of children and young people undertaking such work[11].

This paper reports the findings of a patient/public involvement (PPI) group, where researchers, parents and young people convened to design a study to investigate procalcitonin-assisted decision-making in the management of febrile neutropenia in children undergoing anti-cancer therapy.

Method

A request was made on social media for parents and young people who had experience of childhood cancer therapy to consider taking part in a group to discuss the proposed trial. Volunteers were gathered, and after initially attempting a face to face meeting, a video conference platform (Zoom) was used to overcome geographical barriers to promote inclusiveness and working together. The researchers, all clinical doctors with additional academic roles, all met in one location; the public contributors took part from their own homes. One participant could not get integrated audio working, so joined the conversation via telephone and mute video. The discussion lasted 2h 15 minutes.

The session was structured to introduce febrile neutropenia, the existing evidence for the proposed intervention, and the rationale for a randomised feasibility study (see Box 1 for the initial plan). The PPI group all had knowledge of clinical studies, including trials, in children and young people with cancer. The discussion followed a series of questions about the experience of febrile neutropenia, it's management, the perceived challenges with current approaches and how the study would be best organised to meet these.

The session was video and audio recorded. The entire meeting was transcribed, after audio immersion, and the content thematically studied. Elements of the conversation related specifically to the design and conduct of a study were developed into themes and sub-themes. Elements related to the management of febrile neutropenia were examined in a framework derived from the themes developed in a relevant PhD [12]. Following the meeting, a summary was shared and agreed, and the full report from which this paper is derived was reviewed by the PPI group.

The costs of the group were small; transport costs and light refreshments only for the researchers, and a small fee for the video conferencing platform. The platform and the technologies were already owned by the participants. The participants volunteered their time, in line with their voluntary involvement with similar charitable activities, and did not receive payment.

Confidential: For Review Only

As this was a patient/public engagement group no ethical review was required. This is consistent with the INVOLVE definition of public involvement in research as “research being carried out ‘with’ or ‘by’ members of the public rather than ‘to’, ‘about’ or ‘for’ them” [13] , Despite the lack of a formal requirement for research ethics committee oversight, and ethical approach to such work is necessary. Such an approach has been described[14], and the key elements of a fair choice to partake in the work, appropriate training and support to understand the questions asked, making sure access was as equitable as possible and providing recognition for the work were all considered in this project.

Results

Participants

Four volunteer parents were part of the UK-based PORT (Paediatric Oncology Reference Team) organisation, which consists of parents of children and young people who had experience of childhood cancer. Each was the mother of a child who had undergone cancer treatment; two with leukaemia, two with neuroblastoma. Three of these four parent’s children had died of their disease. A patient who had leukaemia when a teenager also took part in the group, unrelated to the other participants. Of the three researchers, two were higher specialist trainees in paediatric oncology, and one a Consultant, who was the only male in this group. Each member of the group had prior experience with research in children’s cancer beyond participation. The discussions involved descriptions of past experiences of admissions with fever and neutropenia. The experiences were from around 2006-2017. During that time period, there has been a move to some reduction in length-of-stay and marginally more consistency between centres in the UK [15]. Additional comments were added by a fifth PORT parent via email following the video conference.

Study-specific themes

Information regarding the undertaking and conduct of the study was described under three major themes; ‘importance’, ‘how PPI changes researchers’ views’ and ‘practical and ethical’. The theme of ‘importance’ was formed by concepts of ‘medical consequence’, ‘psychological consequence’, ‘impact’, ‘unpredictability’ and ‘frequency of FN’. (See Figure)

Importance: The group members unanimously agreed that the management of episodes of febrile neutropenia was important because of its unpredictability, frequency, and medical, psychological and social (‘impact’) consequences. They described particularly how variation in care across different hospitals was a source of concern to them and consistency would be a positive by-product of undertaking a trial:

> *“we’ve got the 20-odd centres and pretty much everybody follows the same protocols [for anti-cancer treatment]... and I think it would be really reassuring for families if the POSCUs [Paediatric Oncology Shared Care Units] you know if we knew that everybody was doing the same thing”* [P1]

‘How PPI changes researchers’ views’ describes the impact of this work: of how PPI is important in modifying the initial, genuinely held presumptions and beliefs about the best ways to conduct such a

Confidential: For Review Only

trial for families and CYP. These beliefs were drawn from the trial development group, the group of clinicians and researchers involved in designing the trial, which has over a century of experience working in children's cancer in a variety of units and countries and specific expertise in studying supportive care in this group of patients. The impact of the group is seen in in the following aspects:

Allocation. The initial suggestion was for group randomisation, assigned arms to by clinical unit, rather than individual patients. However, the PPI input strongly steered towards individual randomisation, but with each individual receiving the same arm of management throughout the whole trial:

> *with paediatric oncology unlike many other things... everybody is on a trial... everybody... I mean... <snip>... I think everyone just expects that their treatment may be a bit different than everyone else's...* [P2]

> *once you're randomised rather than each time coming in and by randomised each time, you're better off having "this family is procalcitonin, this family is not"* [P1]

Outcome assessment. The group members felt direct measures of patient experience would be important, more than 'quality of life' checklists. They suggested offering a daily experience journal of some form (paper, or 'app' based electronic), and believed this would supplement and enrich the medical data collected, such as admission duration, antibiotic duration, and infective organisms.

> *capturing that idea of burden beyond the hospital based stuff and things that matter to the family,* [P3]

They described how it would be important to measure the extra resources required because of FN admissions. The word 'impact' was felt to capture this rather than 'costs'.

> *impact, because it's not just about extra costs, I mean if you're having to call in grandparents and you're having to call in favours left right and centre, <snip> I mean how many times can you ask the next door neighbours to collect your kids from school* [P1]

Active safety netting. The researchers initially felt the standard approach after discharge of responsibilities passed to the family to 'return if unwell' would be safe and acceptable, but the PPI group thought an active approach to safety-netting was necessary, but should be individually negotiated:

> *I think it needs to be more than just you phone up the hospital if you have any concerns, it should be either somebody coming around or phoning you and saying "Do you have any concerns, do you have any concerns at all" and actually if you say yes …… giving you the option of coming back or having somebody over* [P4]

Gaining consent. The researchers proposed, as with usual practice, the study would be offered once

Confidential: For Review Only

to families when the clinician believed it appropriate. The group members, with their experience of studies and information being exchanged, thought it would be fair to allow people to decline early on, but have the opportunity to join the study if they changed their mind as their treatment journey progressed. They also floated the idea of families approaching clinicians to join, rather than being invited.

> *I don't think I even knew what febrile neutropenia was though, at the time when we were first giving our consent to all the other things? I think that's something that possibly comes with... further on... down the line... even a week or two weeks after you've given all those other consents. Because actually, the other consents are almost live-saving things... whereas this is a real choice... and I think that batching it in with those initial forms of consents is almost taking away your flexibility of trying to consider it whether you want to do it or not* [P4]

> *You might also get people saying 'No' right at the beginning, if it's something they don't have to agree with, and then subsequently further on during their treatment when they can really see the how much of a headache that this can be... [giggles]*

> - *Do you think it's OK then to offer it twice? If somebody says no the first time?* [R1]

> *Yes – I think I do* [P4]

> *Because it's not like chemo A vs chemo B, it's not … it's not crucial like... you can opt in whenever you want* [P2]

The theme of 'practical and ethical considerations' included the ethical aspects of; consent, randomisation, delays introduced by undergoing the trial, equity and equality, and the sharing of trial data. The practical aspects described outcome collection, safety netting, and ensuring the veracity of information collected in the trial.

Randomisation was considered a fair and ethical approach when in clinical equipoise. Along with this, a later discovery one arm proving better than the other was not considered unethical; however if being on the study disadvantaged everyone (for example, by meaning treatment would be delayed while forms were completed) then it would not have been supported. A design which was accessible for the diversity of social, cultural and economic backgrounds of potential participants was essential. Confirmation of the scientific validity of the proposal and clinical equipoise was important to the PPI group. Prior systematic reviews with meta-analysis were felt to be a very comprehensive answer to this question.

Two of the three researchers have a strong interest in individual participant data meta-analysis. A question was asked about data sharing in this context, and the PPI were very enthusiastic in being involved.

Confidential: For Review Only

Definitely share. I think the thing with paediatric oncology is that we do so many international trials together, because thankfully it is rare, but ultimately I think that... [snip] we're here ultimately to try to make things better for kids of the future and if that's part of it, and it is with these meta-analysis, then definitely. [P1]

The group members were concerned about study governance, for example ensuring the veracity of information collected during the study.

"are they [study groups] actually going to tell you the truth?" [P2]

The members were keen to know there would be some ways of determining if the data collected were truthful and accurate: this seems to speak of a greater public awareness being required of the nature of health research governance within the country generally.

An offer for expressions of interest in continuing to engage with the study governance was enthusiastically met by the participants. One of the group has joined the funding application as a co-applicant, and has helped develop the grant application and is planned to be involved in the qualitative data collection and analysis as a co-investigator.

Febrile neutropenia themes

Conversations in these discussions mapped onto the framework proposed by Morgan, developed to understand the decision-making processes involved in manging episodes of febrile neutropenia [12]. The overarching concepts she described were of the quest for certainty, attaining mutual trust, and the potential for realised discretion. These were all strongly endorsed in analysis of the group discussion.

The quest for certainty involves balancing the uncertainty of outcome of each episode of febrile neutropenia, including an appreciation of probability, the use of protocols and guidelines to manage the risk, and acknowledging the adverse elements of hospitalisation. The use of protective isolation, where the child and family are kept in a single room to avoid infections being caught from other hospitalised children, or source isolation, where the child is kept in a single room to avoid an infection they have spreading onwards to others, were viewed particularly negatively.

It was his cupboard – [child] called it his cupboard [P3]

Mutual trust had been a challenge, with the group describing individual health care practitioners in whom they did not place trust, and the reciprocal of this, along with the negating of parental concerns;

the first time that I thought it was that they were taking too much precaution and I would have much preferred him to be at home taking tablets and things... monitored every so often.. whereas the second time I think he needed more than what he was getting... and I think we were right both times actually [P4]

Confidential: For Review Only

The ideal management of an episode of febrile neutropenia was one where safety was assured, hospitalisation was minimised, decisions discussed with families, and support provide at home provided as desired by the family: the potential for realised discretion. The group readily acknowledged the decisions would need to be based on a range of factors, including home-to-hospital distance and the variability between parents and families in self-expressed confidence:

> *my sort of worry is that... the responsibility is even more on the parent as well. .on top of like running the house... and its that sense of responsibility as well... like they're monitoring their child and being responsible for it... and like if something did happen would they feel guilty about it or not?* [P5]

Exploring how the professionals were thinking about the episode, in terms of the likelihood of adverse outcomes and their considerations, was a strength in a shared decision making approach which had been absent in many prior experiences:

> *I think it would be really helpful,* [imitates Dr speaking] *... we think its' like this [left] or we think it's like this [right]... and then chatting... and ….. You know where you are coming from and where there is a difference and you know talking about …* [P2]

> *seem to recall being in negotiations... situations where... ringing [PTC] consultants saying "This is our situation .... can you speak to them and and so on…"* [P4]

Reflections of clinical academics

The group discussions encouraged the three researchers to reflect on their previous approaches to febrile neutropenia and PPI involvement in other studies. The more experienced researchers had undertaken PPI before, but always on a face-to-face basis. The video conferencing allowed for a more diverse group of individuals to undertake the work, with the researchers in the same room on one screen facilitating. The makeshift re-positioning of audio for one participant through the phone served to reduce hierarchies, with collaborative suggestions and problem solving forming an early 'win' for the group. This method worked well with the age and technological skills of this group, but may be less successful if a group with fewer technology skills or younger age were being involved. The protocol changes suggested by the group had been unexpected, as was the emphasis on the emotional burden of physical isolation. The researchers all took away from this experience the value of listening to expert parents and young adults, and considering video or telephone conferencing to allow a greater number and range of people to take part in PPI events.

Discussion

The engagement of a group of parents and an ex-patient who had experience of CYP cancer with researchers developing a study to improve the management of febrile neutropenia led to changes in the proposed design of the trial, and brought out a deeper understanding of the potential concerns of participants in such a study. The wider discussions about the nature of the experience of an episode of febrile neutropenia were congruent with prior work in the field [12] pointing particularly to actively involve parents and young people in sharing decisions about care.

Confidential: For Review Only

The PPI involvement altered how the trial would be structured, randomisation of each child to one of the two management methods through the whole of their anti-cancer treatment, rather than randomising the study sites or the child at each visit (see Box 2 for specific changes). The suggestion of multiple opportunities to be involved in the study was welcome, and congruent with the description of an emerging expertise and empowerment in people through the childhood cancer journey [16]. They discussed practical methods of collecting data which went beyond simple admission statistics and questionnaires, to enrich the information gained in the study. Active follow-up, with healthcare initiated contact with the family, but negotiated in light of their individual family, had not been originally considered by the researchers. The discussion also shed light on the experiences of people in being involved in treatments of episodes of febrile neutropenia, with the ideal being an individualised, negotiated approach within clear, safe, guidance, consistently used across all centres.

The expertise and prior relationships in the group members of similar situations may have enhanced the easy flow of ideas and conversations in this event. All members knew at least one other participant through in-person interactions, in similar group settings or clinical interactions. Ice-breaking activities were extremely brief, as there was little ice to be broken. None of the participants were paid for their time undertaking this work. The INVOLVE guidelines suggest involvement should come with re-imbursement, the group all undertook work with charities related to childhood cancer treatment, research and support and saw this as an extension of their other activities. Future PPI work with similar groups of people would benefit from considering holding the group conversations via a video conferencing platform. The ready availability of web-cams and front-facing cameras on phones, tablet and laptop computers, and the common use of video conversations in work and home life mean these were acceptable methods to have discussions with this group. There are limitations with this approach. It requires a familiarity and access to such equipment, and access to a relatively stable internet connection. This may exclude PPI, particularly young people, from disadvantages backgrounds. It may also be very difficult to use to work with younger children, or older family members, perhaps great-grandparents, who are unfamiliar with video conferencing. If the approach is used, it may be beneficial to have a 'test run' period prior to the meeting to allow any technical challenges to be met; we would suggest a period of time when 'drop in' connections to confirm all is working well would be a sensible way forwards. A backup approach, as simple as a telephone line, is also very helpful.

We used social media (Twitter) to recruit the participants; as the researchers all had prior experience of working with PORT, and 'tagged' them into a post, this may be considered a mixture of open and direct messaging. This type of use has been fairly widely undertaken previously [17] and has advantages and disadvantages. It carries little direct risk, as it doesn't ask for people to engage in discussion in a forum (such as Facebook or Blog comments), but its reach is limited to those who already follow one of the accounts which post, or re-tweet, the invitations. It provided an excellent opportunity to draw in active PPI parent volunteers, but did not attract a large number of young people. Direct advertising of the chance to be involved in the work to young people via other groups, such as Young People's Advisory Groups hosted by organisations such as the National Cancer Research Institute or Clic-Sergant, or advertising through the Teenage Cancer Trust, may have meant more than one young person was involved.

The findings of this study have immediately influenced an application for a feasibility study of procalcitonin guided management of febrile neutropenia. They will also influence the ongoing

Confidential: For Review Only

development of clinical practice through dissemination through the children's and young people's professional network groups. The participants in this group have expressed a wish to be part of the steering committee of a trial addressing this issue, and the ongoing study development will also seek further young people to be involved, following INVOLVE guidelines[17 18]. One of the group members has joined the study as a co-applicant, developing the grant, and plans to be involved as a co-investigator..

Figure legend

Interaction of themes and sub-themes

Funding statement

This research received no specific grant from any funding agency in the public, commercial or not-for-profit sectors. BP was supported by an NIHR Post-doctoral fellowship: grant number PDF2014-10872.

Competing Interests

There are no competing interests to declare.

Contributorship

This study was conceived by BP and SD, and developed with the assistance of JM. The audio was transcribed and analysed by BP initially with input from JM and SD. BP drafted the paper, and was critically revised and developed by JM and SD. The PPI group read and agreed with the content of the paper. The authors very gratefully acknowledge their input into this specific work.

Box 1: Original trial design

Site-randomised trial (randomising by hospital) using cluster or step-wedge approach

Use of single quality of life questionnaire at discharge

Patient contact for trial purposes only to occur while in-patient – not after discharge

Antibiotic decision making on procalcitonin measurements and clinical judgement without family involvement

Box 2:Changes following consultation

Individual patient randomised trial (randomising by patient, not by episode)

Consent permissible at any point during the cancer journey while still 'at risk'

Confidential: For Review Only

Richer patient experience measures – not just patient QoL but family experience and their costs to be captured

Active follow-up after discharge

Explicitly encouraging shared decision making and sharing of results with families to decide antibiotic use

Confidential: For Review Only

References

1. Gatta G, Botta L, Rossi S, et al. Childhood cancer survival in Europe 1999–2007: results of EUROCARE-5—a population-based study. *The Lancet Oncology* 2014;15(1):35-47. doi: https://doi.org/10.1016/S1470-2045(13)70548-5

2. Creutzig U, Zimmermann M, Reinhardt D, et al. Early deaths and treatment-related mortality in children undergoing therapy for acute myeloid leukemia: analysis of the multicenter clinical trials AML-BFM 93 and AML-BFM 98. *J Clin Oncol* 2004;22(21):4384-93. doi: 22/21/4384 [pii]

10.1200/JCO.2004.01.191 [published Online First: 2004/10/30]

3. Hassan H, Rompola M, Glaser AW, et al. Validation of a classification system for treatment-related mortality in children with cancer. *BMJ Paediatrics Open* 2017;1(1):e000082. doi: 10.1136/bmjpo-2017-000082

4. Morgan JE, Cleminson J, Stewart LA, et al. Meta-ethnography of experiences of early discharge, with a focus on paediatric febrile neutropenia. *Supportive Care in Cancer* 2018;26(4):1039-50. doi: 10.1007/s00520-017-3983-2

5. Lehrnbecher T, Robinson P, Fisher B, et al. Guideline for the Management of Fever and Neutropenia in Children With Cancer and Hematopoietic Stem-Cell Transplantation Recipients: 2017 Update. *J Clin Oncol* 2017;35(18):2082-94. doi: 10.1200/JCO.2016.71.7017

6. Phillips RS, Sung L, Amman RA, et al. Predicting microbiologically defined infection in febrile neutropenic episodes in children: global individual participant data multivariable meta-analysis. *Br J Cancer* 2016;114(6):623-30. doi: 10.1038/bjc.2016.28

7. Haeusler GM, Thursky KA, Mechinaud F, et al. Predicting Infectious ComplicatioNs in Children with Cancer: an external validation study. *British Journal of Cancer* 2017;117(2):171-78. doi: 10.1038/bjc.2017.154

8. Haeusler GM, Carlesse F, Phillips RS. An Updated Systematic Review and Meta-Analysis of the Predictive Value of Serum Biomarkers in the Assessment of Fever during Neutropenia in Children with Cancer. *Pediatr Infect Dis J* 2013;32(10):e390-6. doi: 10.1097/INF.0b013e31829ae38d [published Online First: 2013/05/16]

9. de Jong E, van Oers JA, Beishuizen A, et al. Efficacy and safety of procalcitonin guidance in reducing the duration of antibiotic treatment in critically ill patients: a randomised, controlled, open-label trial. *The Lancet Infectious Diseases* 2016;16(7):819-27. doi: https://doi.org/10.1016/S1473-3099(16)00053-0

10. Stocker M, van Herk W, el Helou S, et al. Procalcitonin-guided decision making for duration of antibiotic therapy in neonates with suspected early-onset sepsis: a multicentre, randomised controlled trial (NeoPIns). *The Lancet* 2017;390(10097):871-81. doi: https://doi.org/10.1016/S0140-6736(17)31444-7

11. Bird D, Culley L, Lakhanpaul M. Why collaborate with children in health research: an analysis of the risks and benefits of collaboration with children. *Archives of disease in childhood - Education & practice edition* 2013;98(2):42-48. doi: 10.1136/archdischild-2012-303470

12. Morgan JE, Phillips B, Stewart LA, et al. Quest for certainty regarding early discharge in paediatric low-risk febrile neutropenia: a multicentre qualitative focus group discussion study involving patients, parents and healthcare professionals in the UK. *BMJ Open* 2018;8(5):e020324. doi: 10.1136/bmjopen-2017-020324

13. INVOLVE. What is public involvement in research? 2018 [Available from: http://www.invo.org.uk/find-out-more/what-is-public-involvement-in-research-2 accessed October 2018.

14. Mitchell SJ, Slowther A-M, Coad J, et al. Ethics and patient and public involvement with children and young people. *Archives of disease in childhood - Education & practice edition* 2018 doi: 10.1136/archdischild-2017-313480

15. Morgan JE, Phillips B. Winter 2017 Children's Cancer and Leukaemia Group febrile neutropenia audit. *Arch Dis Child* 2018 doi: 10.1136/archdischild-2018-315249

Confidential: For Review Only

16. Mu PF, Lee MY, Sheng CC, et al. The experiences of family members in the year following the diagnosis of a child or adolescent with cancer: a qualitative systematic review. *JBI Database System Rev Implement Rep* 2015;13(5):293-329. doi: 10.11124/jbisrir-2015-1698
17. INVOLVE. Guidance on the use of social media to actively involve people in research: INVOLVE, 2017.
18. INVOLVE. Involving children and young people in research: top tips and essential key issues for researchers 2016: INVOLVE, 2016.

Confidential: For Review Only

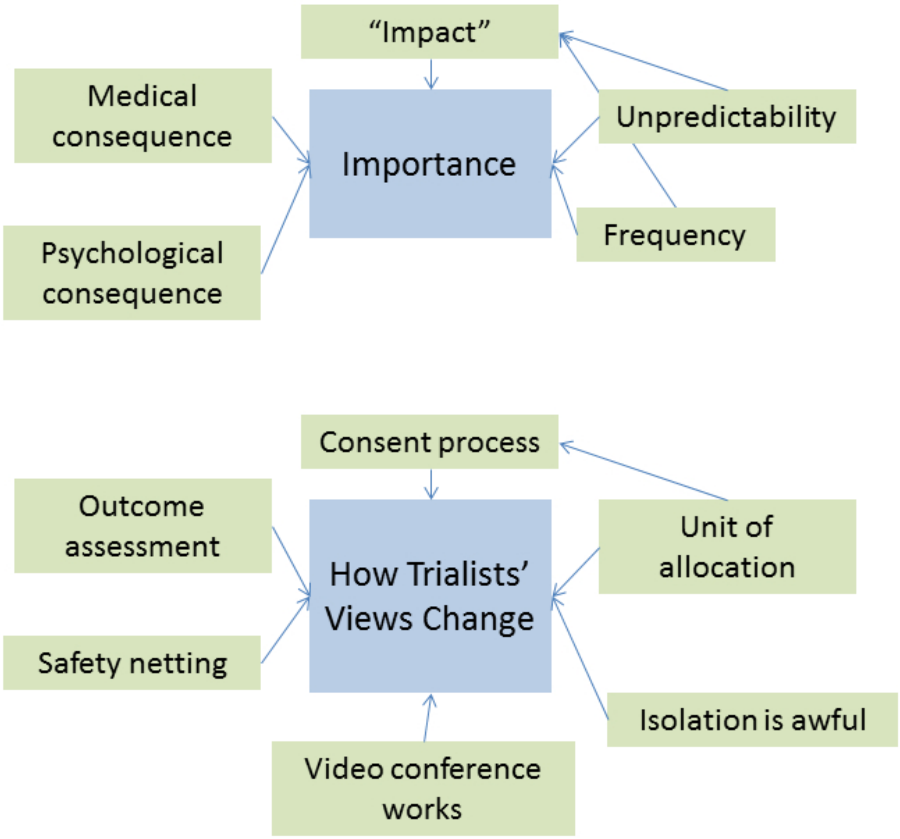

60x55mm (300 x 300 DPI)

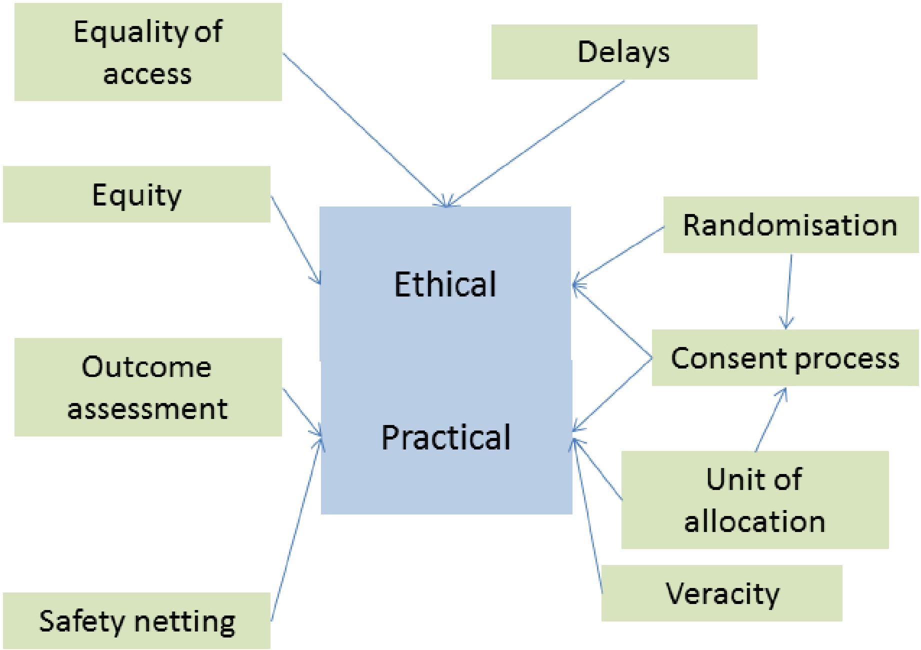

58x43mm (300 x 300 DPI)
